# Supplementary material for: Standard and reference‐based conditional mean imputation
Source: Pharm Stat. 2022 May 19;21(6):1246–57. doi: 10.1002/pst.2234 (PMC9790242; doi:10.1002/pst.2234)
Supplement: Supplementary file 1 — Appendix S1 Supporting Information [file PST-21-1246-s001.pdf]

Supplementary materials for the manuscript "Standard and reference-based conditional mean imputation": Additional details on bootstrap methods

Bootstrap confidence intervals (CI) and tests for the treatment effect

A large number of bootstrap methods for CI estimation and testing have been proposed in the literature. We first discuss methods based on the bootstrap standard error and the normal approximation and percentile bootstrap methods [1, 2].

Denote the treatment effect estimates from B bootstrap samples by $\hat{\theta}^*_b$ $(b = 1, \ldots, B)$. The *bootstrap standard error* $\hat{se}_{boot}$ is defined as the empirical standard deviation of the bootstrapped treatment effect estimates. The corresponding two-sided normal approximation $(1-\alpha)$ CI is defined as $\hat{\theta} \pm z^{1-\alpha/2} \cdot \hat{se}_{boot}$ where $\hat{\theta}$ is the treatment effect estimate in the original data set, i.e. $\hat{\theta} = \hat{\theta}_{CMI}$. Tests of the null hypothesis $H_0 : \theta = \theta_0$ are then based on the Z-score $Z = (\hat{\theta} - \theta_0)/\hat{se}_{boot}$ using a standard normal approximation.

Alternatively, *percentile bootstrap* methods can be used for inference: For this method, a two-sided $(1-\alpha)\%$ CI is defined as the interval from the $((B+1)\alpha/2)^{th}$ to the $((B+1)(1-\alpha/2))^{th}$ ordered bootstrap treatment effect estimate [2, page 202f]. Typically, B is chosen such that $(B+1)\alpha/2$ is an integer value. If this is not the case, the corresponding empirical bootstrap quantiles can be calculated via interpolation. The advantage of the percentile method over the standard normal approximation is that it is also valid if the treatment effect estimator itself is not normally distributed around the population treatment effect but if a monotone transformation exists which normalizes the estimator [1, page 173f]. For example, this is the case for t-distributed test statistics.

A *one-sided* bootstrap p-value for a test of the null hypothesis $H_0 : \theta = \theta_0$ versus the alternative $H_1 : \theta > \theta_0$ can be defined via inversion of the percentile CI. If exactly one of the bootstrap samples results in an estimate of θ_0, then the p-value is defined as $\hat{p} = (\#\{\hat{\theta}^*_b < \theta_0\} + 1)/(B+1)$ [1, page 214-215; adding +1 to comply with the percentile bootstrap definition of 2]. Otherwise, one needs to numerically determine to which quantile of the bootstrap distribution θ_0 corresponds. The *one-sided* test in the other direction can be defined in the same way, and the corresponding two-sided p-value is defined as 2 times the lower of the two one-sided p-values.

More complex bootstrap confidence intervals also exist and the BCa (bias-corrected and accelerated) CI is often recommended [3]. This would be also possible but not trivial in our setting: Typically, a is estimated based on the skewness of the score function [2, page 205] (which is not available in closed form in our setting) or on a jackknife approximation to it [3] (which is computationally intensive in our setting).

Permutation tests and associated bootstrap tests which draw bootstrap samples under the null hypothesis of equality between the two randomized treatment groups [1, chapters 15 and 16; 2, chapter 4] could also be applied but have not been explored in our setting.

How many bootstrap samples?

As a general rule of thumb, Davison and Hinkley [2, pages 156 and 202] advise to use $B = 999$ or more for CI estimation and testing. However, as illustrated below, substantially larger values of B may be required for accurate estimation of p-values. Let $\hat{p}_\infty$ denote the one-sided p-value that would be obtained under the theoretical bootstrap distribution (including all possible bootstrap samples) and $\hat{p}_B$ the corresponding p-value based on B bootstrap samples. We use the following approximate distributions for $\hat{p}_B$:

- $\hat{p}_B \sim \Phi(\Phi^{-1}(\hat{p}_\infty)/\sqrt{Z/(B-1)})$ with $Z \sim \chi^2(df = B-1)$ for the normal approximation
- $\hat{p}_B \sim (Z+1)/(B+1)$ with $Z \sim Bin(B, \hat{p}_\infty)$ for the inverted bootstrap percentile method

Based on these approximations, the probabilities below were evaluated:

**Method**	**B=999**	**B=9,999**	**B=99,999**
**Normal approximation**			
95% range for $\hat{p}_B$ if $\hat{p}_\infty = 2.5\%$	2.02% to 3.02%	2.34% to 2.66%	2.45% to 2.55%
Probability of $\hat{p}_B \leq 2.5\%$ if $\hat{p}_\infty = 2\%$	98.37%	100.00%	100.00%
Probability of $\hat{p}_B > 2.5\%$ if $\hat{p}_\infty = 3\%$	96.42%	100.00%	100.00%
**Percentiles method**			
95% range for $\hat{p}_B$ if $\hat{p}_\infty = 2.5\%$	1.70% to 3.60%	2.21% to 2.82%	2.40% to 2.60%
Probability of $\hat{p}_B \leq 2.5\%$ if $\hat{p}_\infty = 2\%$	84.66%	99.97%	100.00%
Probability of $\hat{p}_B > 2.5\%$ if $\hat{p}_\infty = 3\%$	84.54%	99.88%	100.00%

References

[1] Bradley Efron and Robert J Tibshirani. *An introduction to the bootstrap.* CRC press, 1994.

[2] Anthony C Davison and David V Hinkley. *Bootstrap methods and their application.* Cambridge University Press, 1997.

[3] James Carpenter and John Bithell. Bootstrap confidence intervals: when, which, what? a practical guide for medical statisticians. *Statistics in Medicine*, 19(9):1141–1164, 2000.
